# Multi-nuclear magnetic resonance spectroscopy: state of the art and future directions

**DOI:** 10.1186/s13244-022-01262-z

**Published:** 2022-08-17

**Authors:** Yi Wei, Caiwei Yang, Hanyu Jiang, Qian Li, Feng Che, Shang Wan, Shan Yao, Feifei Gao, Tong Zhang, Jiazheng Wang, Bin Song

**Affiliations:** 1 Department of Radiology, West China Hospital, Sichuan University, No. 37, Guoxue Alley, Chengdu, 610041 People’s Republic of China; 2 Clinical & Technical Support, Philips Healthcare, Beijing, China; 3 Department of Radiology, Sanya People’s Hospital, Sanya, China

**Keywords:** Hyperpolarization, Multi-nuclear magnetic resonance, Magnetic resonance spectroscopic imaging, Clinical application

## Abstract

With the development of heteronuclear fluorine, sodium, phosphorus, and other probes and imaging technologies as well as the optimization of magnetic resonance imaging (MRI) equipment and sequences, multi-nuclear magnetic resonance (multi-NMR) has enabled localize molecular activities in vivo that are central to a variety of diseases, including cardiovascular disease, neurodegenerative pathologies, metabolic diseases, kidney, and tumor, to shift from the traditional morphological imaging to the molecular imaging, precision diagnosis, and treatment mode. However, due to the low natural abundance and low gyromagnetic ratios, the clinical application of multi-NMR has been hampered. Several techniques have been developed to amplify the NMR sensitivity such as the dynamic nuclear polarization, spin-exchange optical pumping, and brute-force polarization. Meanwhile, a wide range of nuclei can be hyperpolarized, such as ^2^H, ^3^He, ^13^C, ^15^ N, ^31^P, and ^129^Xe. The signal can be increased and allows real-time observation of biological perfusion, metabolite transport, and metabolic reactions in vivo, overcoming the disadvantages of conventional magnetic resonance of low sensitivity. HP-NMR imaging of different nuclear substrates provides a unique opportunity and invention to map the metabolic changes in various organs without invasive procedures. This review aims to focus on the recent applications of multi-NMR technology not only in a range of preliminary animal experiments but also in various disease spectrum in human. Furthermore, we will discuss the future challenges and opportunities of this multi-NMR from a clinical perspective, in the hope of truly bridging the gap between cutting-edge molecular biology and clinical applications.

## Key points

- The multi-NMR by HP has been applied in many diseases. It has allowed for real-time assessment of human metabolism in vivo and can promote a shift from morphological to molecular level.
- Work is ongoing in clinical translation of multi-NMR, with numerous spectrum of human diseases, and bridging the gap between the molecular level imaging and clinical applications will foster and introduce future opportunities in this field.

## Background

Since 1938, the phenomenon of NMR discovered by Rabi when sent a beam of molecules through magnetic field and found that they could be made to emit radio waves at specific frequencies, great advances in the research and clinical applications of magnetic resonance (MR) imaging have been obtained and leaved remarkable contributions in human health [1]. At present, the development of medical imaging is not only confined to observation of lesions from morphological changes, but also toward a more in-depth comprehensive assessment of biological behavior, pathophysiological, and metabolic changes, and thus to achieve a more precise personalized medicine. With the development of MR hardware (magnets, gradients, radiofrequency coils) and software (pulse sequences, data reconstruction algorithms), the clinical application of multi-nuclear magnetic resonance spectroscopy provides the possibility of real-time dynamic visualization of the metabolic changes and specific substrate to metabolic conversion of the lesion in vivo, which also pushes the MR imaging into a more in-depth molecular level [2].

In theory, any nucleus that can be detected by NMR can be imaged with MR imaging. ^1^H is the mostly used atom because of the high concentration in human body and large value of the gyromagnetic ratio which enables to achieve high-resolution T1- or T2-weighted MR images. However, the complicated background signal, signal overlaps, and the ability to only reflect a few molecules such as such as choline, creatine, NAA, glycine, myo-inositol, lactate, alanine, and acetate for ^1^H also limit its application in the real-time metabolic activity. Using of higher magnetic fields, developing of new detectors, increasing the signal with hyperpolarization, and obtaining the signal with ultra-fast pulse sequences make the other nucleus imaging spectroscopy like ^2^H, ^13^C, ^15^ N, ^18^F, ^23^Na, ^31^P, and ^129^Xe come true [3–5]. These multi-NMR spectroscopies provide the ability to insight the multiple metabolic activities and reflect the real metabolism in vivo and have the potential to really bridge the gap between the cutting-edge molecular biology, biochemistry, and metabolism research.

In this review, we will provide an overview of the clinical application of multi-nuclear magnetic resonance spectroscopy in the various biologic processes with different lesions. In addition, we will also discuss the future challenges and opportunities of this novel imaging technique from a clinical perspective, hoping to real bridge the gap between the cutting-edge molecular biology and clinical applications.

## Nervous system

**Fig. 1:**
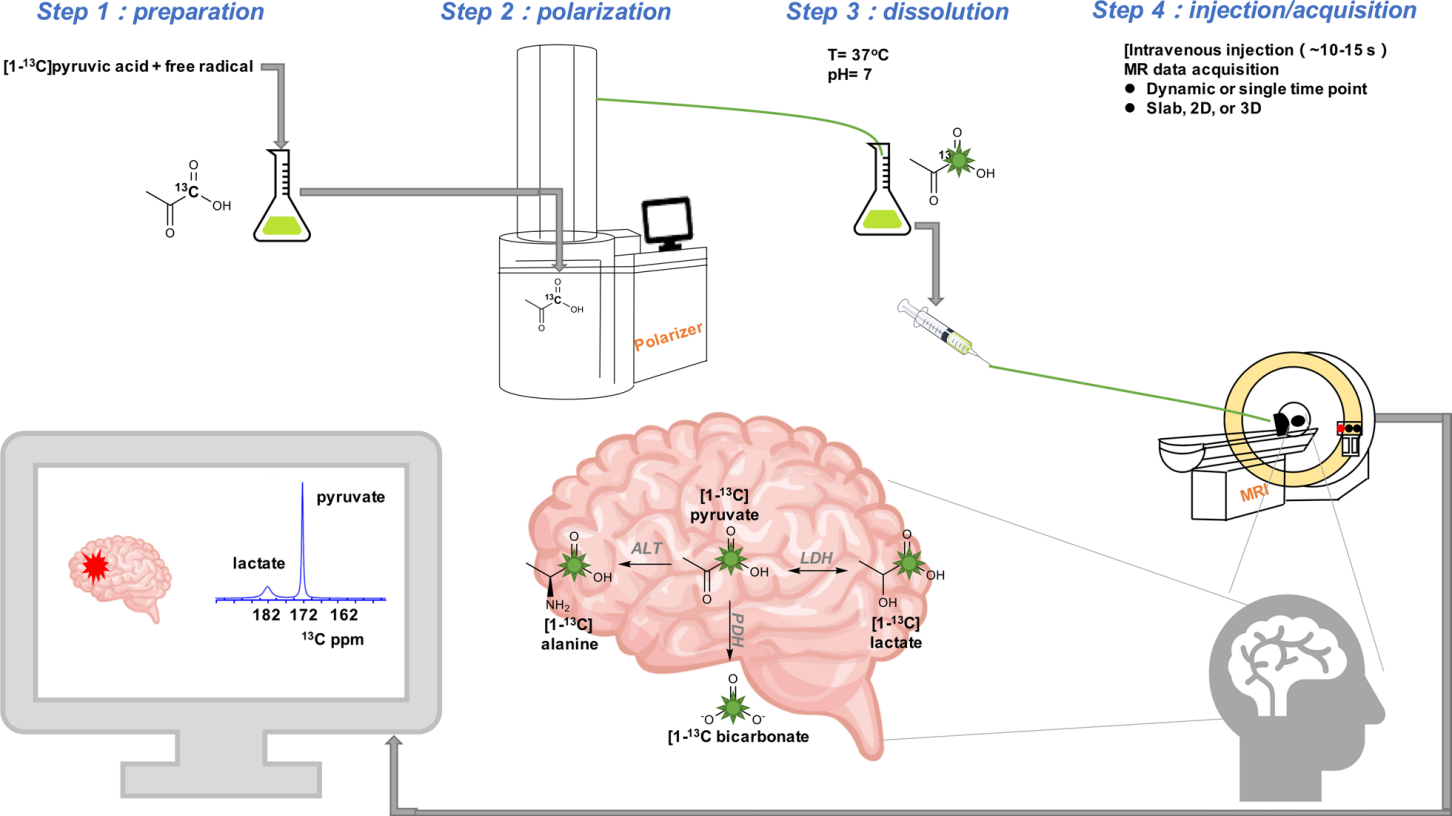
The workflow of hyperpolarized carbon-13 MRI in biomedical imaging

Metabolic abnormalities are key factors in many neurological disorders. The ability to accurately measure this metabolic damage could improve the detection and diagnosis of disease progression and facilitate the development of new treatments accordingly. Hyperpolarized (HP)-carbon-13 magnetic resonance spectroscopic imaging (MRSI) is an emerging imaging technique that allows the noninvasive, real-time measurement of enzyme activity in living organisms (Fig. 1). To date, this metabolic imaging method has been used mainly in the fields of cancer and cardiology and has recently begun to be used to study neurological diseases [6, 7]. HP-^13^C MRSI has promising applications in neurodegenerative diseases, traumatic brain injury, stroke, and brain tumors [8].

### Neurodegenerative diseases

Mononuclear phagocytes (MPs) play a crucial role in the progression of multiple sclerosis (MS) and other neurodegenerative diseases [9–11]. Following activation and subsequent differentiation toward a proinflammatory phenotype, abnormalities in MPs metabolism occur, leading to increased glycolysis and lactate production. HP-^13^C MRSI is a clinically translatable imaging modality that allows for the noninvasive detection of metabolic pathways in real time. ^13^C MRSI not only tracks the Warburg effect within the tumor but also allows for a realistic neurological inflammatory response. The significant increase in HP-[1-^13^C]-pyruvate to HP-[1-^13^C]-lactate conversion, on the one hand, indicated the presence of a high density of proinflammatory MPs. On the other hand, it suggested that the increase in HP-[1-^13^C]-lactate may be mediated by upregulation of pyruvate dehydrogenase (PDH) kinase-1 in activated MPs leading to regional PDH inhibition [12]. Overall, the preclinical results of the cuprizone model demonstrated the potential of ^13^C MRSI of HP-[1-^13^C]-pyruvate as a neuroimaging method for the assessment of inflammatory lesions. This method could prove useful not only for multiple sclerosis, but also for other neurological disorders with an inflammatory component in the future.

### Traumatic disease

**Fig. 2:**
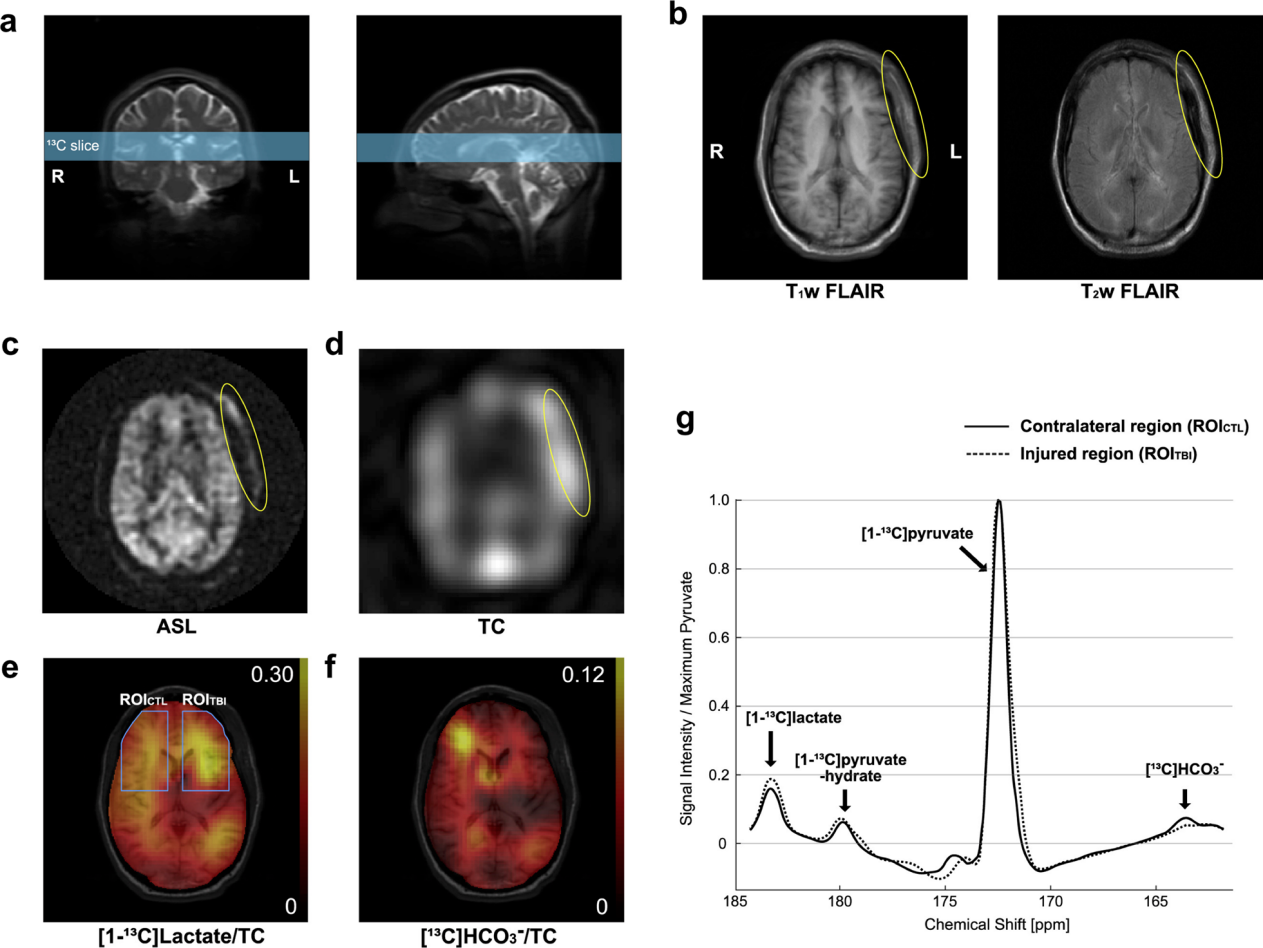
Metabolism of acute traumatic brain injury (TBI) imaged by hyperpolarized 13C-pyruvate. The patient (35 years old, male), injured on the left frontal scalp, was imaged 28 h after the injury. **a** An axial slice that includes the injured region was prescribed for 13C imaging. **b** T1-weighted and T2-weighted 1 H FLAIR and (**c**) ASL images showed swelling and increased perfusion in the injured site outside the skull (yellow circle), whereas no cerebral contusion, hemorrhage or hypoperfusion were detected. **d** Region with increased total hyperpolarized 13C signal image matched to the scalp edema. **e** Increased [1-13C]lactate conversion and (**f**) decreased [13C]HCO3 – production were detected in the brain tissues of the injured hemisphere. **g** Averaged spectra over the injured brain region and the contralateral normal-appearing brain region. Reproduced with permission from Elsevier Ltd (License Number: 5324100264979). iScience. 2020 Nov 30;23(12):101,885

Traumatic brain injury (TBI) can lead to disturbances in energetic metabolism in the brain but monitoring of metabolic activity is currently achieved mainly by microdialysis and ^18^F-fluorodeoxyglucose (^18^F-FDG) positron emission tomography (PET) [13]. Proton magnetic resonance spectroscopy (^1^H MRS) also detected focal or systemic elevated lactate and related PDH caused by primary or secondary injured inflammation [14, 15]. HP-^13^C MRSI could be used as a direct, rapid, and noninvasive method to explore the effects of TBI on energetic metabolism in the brain [16, 17]. In rats with moderate TBI induced by control cortical impingement on one cerebral hemisphere, measured by injection of HP-[1-^13^C]-pyruvate, the injured side of the brain was found to produce a ^13^C-bicarbonate signal 24 ± 6% lower than the injured side, while the HP-bicarbonate-to-HP-lactate ratio was 33 ± 8% lower than that of the injured side [16]. In controls, there were no significant differences in signals between the two cerebral sides. The results suggest that mitochondrial pyruvate metabolism is impaired, resulting in reduced aerobic respiration at the injury site after TBI. Clinically, HP-[1-^13^C]-pyruvate MRSI is used to image patients with acute mild TBI several days after head trauma, but with no obvious anatomic changes. One patient showed high levels of lactic acid production at the injured site, and both patients showed a notable reduction in bicarbonate production [17] (Fig. 2). This study indicates using HP-pyruvate feasibly to image the metabolic changes in TBI patients and proves the transformability and sensitivity of this technology to the changes in cerebral metabolism after mild TBI.

### Stroke

**Fig. 3:**
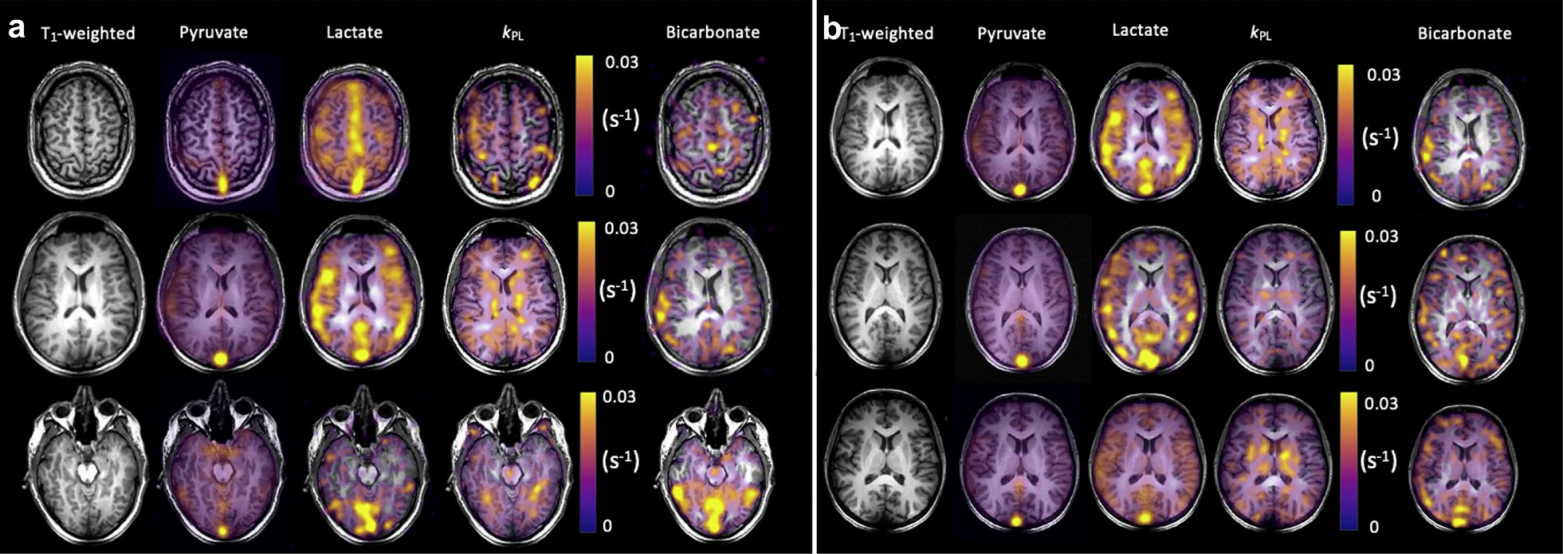
Iterative Decomposition with Echo Asymmetry and Least squares estimation (IDEAL) spiral 13C imaging demonstrating metabolite distribution in the healthy human brain. NeuroImage Volume 189, 1 April 2019, Pages 171–179

Ischemic stroke is a progressive process characterized by impaired but reversible damage to the metabolic activity of the penumbra tissue so that if blood flow is restored, full recovery of function is possible. Currently, ischemic cases are identified by a mismatched area between perfusion and diffusion on MRI. Conventional perfusion and diffusion proton MR images have a large background signal, and the commonly used contrast injection is an invasive method, but many patients with the renal injury cannot use contrast agents and results have shown that gadolinium contrast agents can cause nephrogenic systemic fibrosclerosis. Due to the high rate of glucose extraction in the penumbra, characterized by anaerobic glycolysis and subsequent increased lactate production, this can be detected by the altered metabolism of HP-^13^C-pyruvate. HP-^13^C MRI may therefore have an important role in understanding the development and response of the ischemic putative difficult zone in stroke and is also translatable to concurrent studies of metabolism and perfusion in humans [18] (Fig. 3). In preclinical trials, an endothelin-1-induced ischemic stroke model was used to assess metabolic alterations after acute ischemic stroke in rats [19]. The results showed increased total lactate production in the ischemic hemispheric dark zone compared to the contralateral brain. This was the result of a combination of increased pyruvate in the blood and lactate dehydrogenase (LDH)-mediated lactate production. When estimating penumbra viability, measures of penumbra metabolic activity may be more sensitive than conventional imaging techniques. Likewise, since the ^129^Xe signal is proportional to cerebral blood flow as a perfusion missing agent for cerebral blood flow, HP-^129^Xe MRI can also be used for the study of ischemic semi-dark areas as a perfusion missing agent of cerebral blood flow. The high lipid solubility, lack of biological tissue background signals, and high sensitivity of HP-^129^Xe allow the detection of HP-^129^Xe signals even at low concentrations, which is advantageous for the identification of under-perfused brain tissue. Using HP-^129^Xe MRI, Zhou et al. [20] successfully detected a local decrease in cerebral blood flow and studied ischemic lesions in the core of stroke by performing a permanent middle cerebral artery occlusion on the right brain of rats. This demonstrated that HP-^129^Xe MRI can be used as a complementary tool to conventional MRI to study the pathophysiology of cerebral under perfusion.

### Brain tumor

The current “open questions” in neuro-oncology imaging are to evaluate tumor response during and after treatment on clinically relevant timescales, to differentiate between recurrent or residual tumors and treatment-related changes with high specificity, to classify tumor pathological aberrations at the molecular level, and to predict tumor response to treatment. Metabolic biomarkers could potentially address these issues and could in principle monitor response before genetic alterations or more delayed morphological changes or recurrence.

Hydrogen is the basic building block of biological organic molecule, and therefore, deuterium (hydrogen 2 (^2^H)) is characterized by nondestructive labeling, labeling flexibility, and potential adaptability. At present, in the early stages of research, animal studies have shown that ^2^H MRSI and MRI can be used to quantify ^2^H-labeled glucose metabolism in tumors [21–23].

**Fig. 4:**
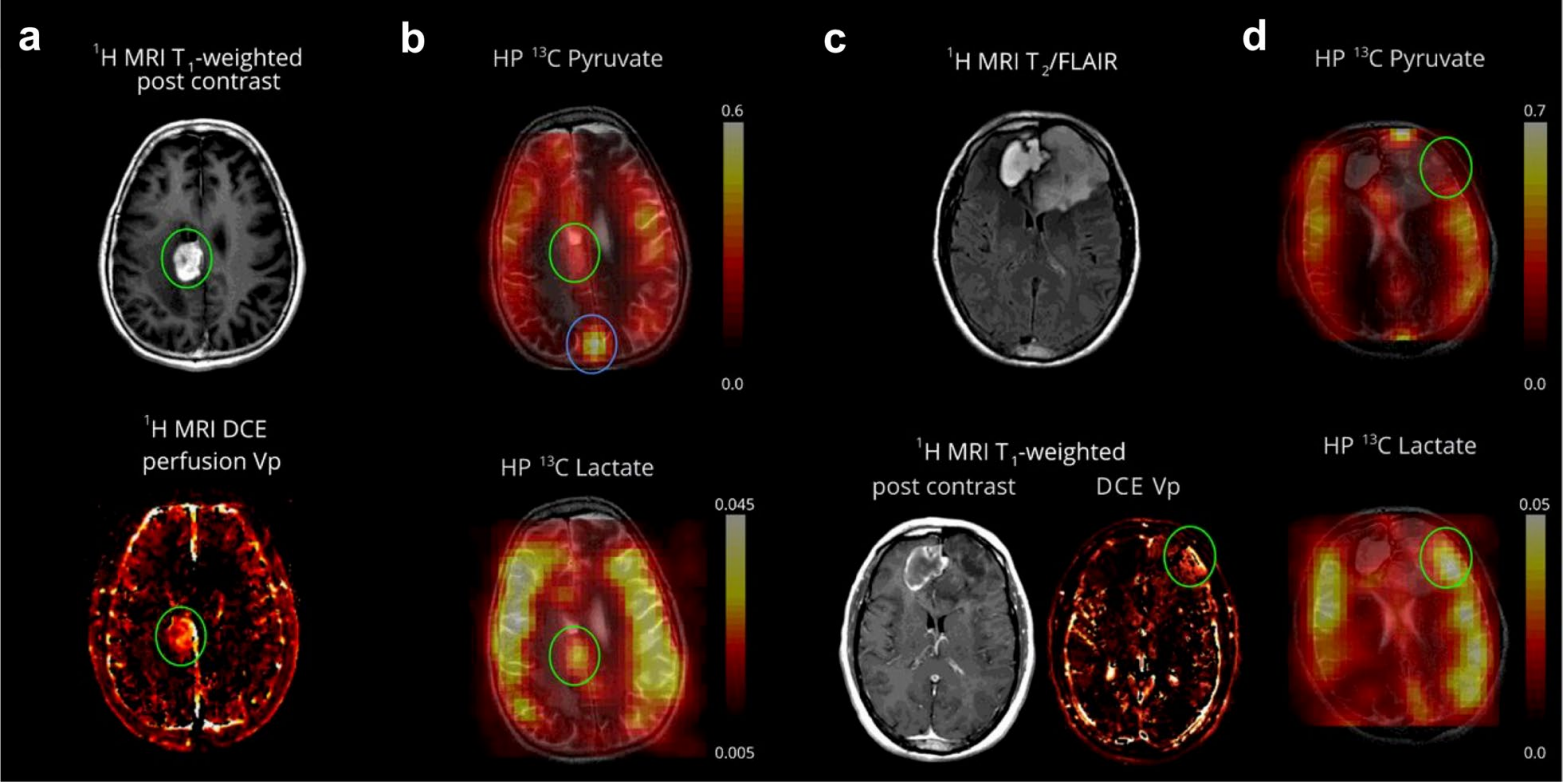
Pyruvate metabolism in a recurrent glioblastoma and treatment-related changes (Patient 2, a and b) and anaplastic oligodendroglioma (Patient 1, c and d). An enhancing mass is located in the right cingulate gyrus (green oval) (**a**). The hyperpolarized pyruvate map shows high signal corresponding to the cortex/juxtacortical regions and superior sagittal sinus. The volume normalized pyruvate signal in the lesion is 1.3-fold higher than the entire brain. The HP lactate map shows mildly elevated signal corresponding to the lesion, similar to background lactate production by the brain. The volume normalized lactate signal in the lesion is 1.0-fold of the lactate over the entire brain (cortex and white matter) (**b**). The T2-FLAIR image shows an expansile left frontal mass and contracting right frontal hematoma. The left frontal mass has minimal, patchy enhancement and a small component of elevated plasma volume (green oval) visualized by DCE MRI (**c**). The HP pyruvate map shows high signal corresponding to the cortex/juxtacortical regions and superior sagittal sinus, but relatively lower signal corresponding to the small hyperperfused component. The HP lactate map shows mildly elevated signal corresponding to the hyperperfused component, similar to background lactate production by the brain (**d**). Cancer Res. 2018;78(14):3755–3760

HP-[1-^13^C]-pyruvate MRI is a novel method for demonstrating energetic metabolism in the human brain and brain tumors, in contrast to the clinical needs that cannot be met by current conventional imaging techniques [24–28] (Fig. 4). HP-pyruvate MRSI was a valuable tool for monitoring the phosphatidylinositol 3-kinase inhibitor LY294002 in mouse models of malignant glioma and breast cancer, where inhibition of the phosphatidylinositol 3-kinase pathway led to reduced LDH activity, thereby reducing the signal for HP conversion of pyruvate to lactate [29]. Park et al. also used HP-^13^C-pyruvate MRSI to assess the prognosis of a rat glioma model [24]. The signal-to-noise ratio of ^13^C-pyruvate and its metabolic^13^C-lactate were both significantly higher than that of normal brain tissue, where the blood–brain barrier (BBB) was able to restrict the entry of pyruvate into brain cells, whereas the disruption of the BBB in gliomas resulted in the smooth entry of ^13^C-pyruvate into brain tissue, which had lagged its research in oncology and cardiology. Nevertheless, Hurd et al. found that injections of [1-^13^C]-ethyl pyruvate (a lipophilic pyruvate derivative) were able to cross the BBB faster than pyruvate and had a significantly higher polarization ratio than total carbon in brain tissue [25], opening a new direction for HP MRSI in the brain. In preliminary studies performed in patients with primary brain tumors, HP-[1-^13^C]-pyruvate rapidly crossed the BBB and was converted to the metabolites lactate and bicarbonate. Non-tumor areas showed a high bicarbonate signal, while tumor areas had a low bicarbonate signal, suggesting that this technique may be valuable for exploring mitochondrial oxidative metabolism and its alterations in brain tumors [26]. Another recent study similarly formalized the feasibility of obtaining high-quality metabolite maps in patients with primary or metastatic brain tumors [27]. These initial clinical studies demonstrate the feasibility of HP-[1-^13^C]-pyruvate MRI for cerebral metabolism assessment.

## Genitourinary system disease

### Tumors

In prostate cancer, antisense oligonucleotides targeting monocarboxylate transporter (MCT)-4 that inhibit lactate secretion may be useful in treating castration-resistant prostate cancer and other cancers because they can interfere with the reprogrammed energetic metabolism of cancer, an emerging hallmark of cancer [30]. Recent preclinical experiments demonstrated that MCT1 regulated pyruvate transport into the cell, MCT4 regulated lactate transport outside the cell body and MCT1 expression had a strong positive correlation with Gleason grade of prostate cancer [31]. Similarly, Keshari et al. used the HP-^13^C-pyruvate co-reactor to study renal cancer cells and found that metabolic pathways could be observed in real time and that changes in the microenvironment caused by oxidation, flux, pH, and substrate could be controlled, revealing the importance of MCT4 transport in disease onset and progression and that MCT4-mediated regulation of lactate transport could be detected by HP-^13^C MRSI [32]. Sriram et al. found that in malignant cells, MCT4 transport of lactate to the extracellular compartment was increased, so the ratio of intracellular to extracellular lactate predicted the aggressiveness of tumor cells [33]. Another research confirmed by HP-^13^C-pyruvate metabolism in in vivo renal tumor tissue that, compared with benign renal tumors, approximately 70–80% of renal cell carcinomas (RCC) had increased lactate production and extracellular translocation was increased, and its aberrant metabolism was mainly caused by increased expression of LDHA and MCT4 [34]. Therefore, benign renal tumors can be differentiated from RCC by assessing the rate of HP-[1-^13^C]-pyruvate-to-lactate conversion and extracellular transport of lactate. In addition, HP-[1-^13^C]-pyruvate MRI can noninvasively assess the aggressiveness of RCC. Sriram et al. [35] found that the expression of LDHA-catalyzed conversion of pyruvate to lactate varied among tumor lines, and HP- [1-^13^C]-pyruvate MRI and diffusion-weighted imaging were used to differentiate between benign renal tumors and renal cell carcinomas by tumor [1-^13^C]-pyruvate conversion rate, LDHA expression rate, and [1-^13^C]-lactate apparent diffusion coefficient values to assess the aggressiveness of renal tumors and to guide the corresponding clinical treatment.

**Fig. 5:**
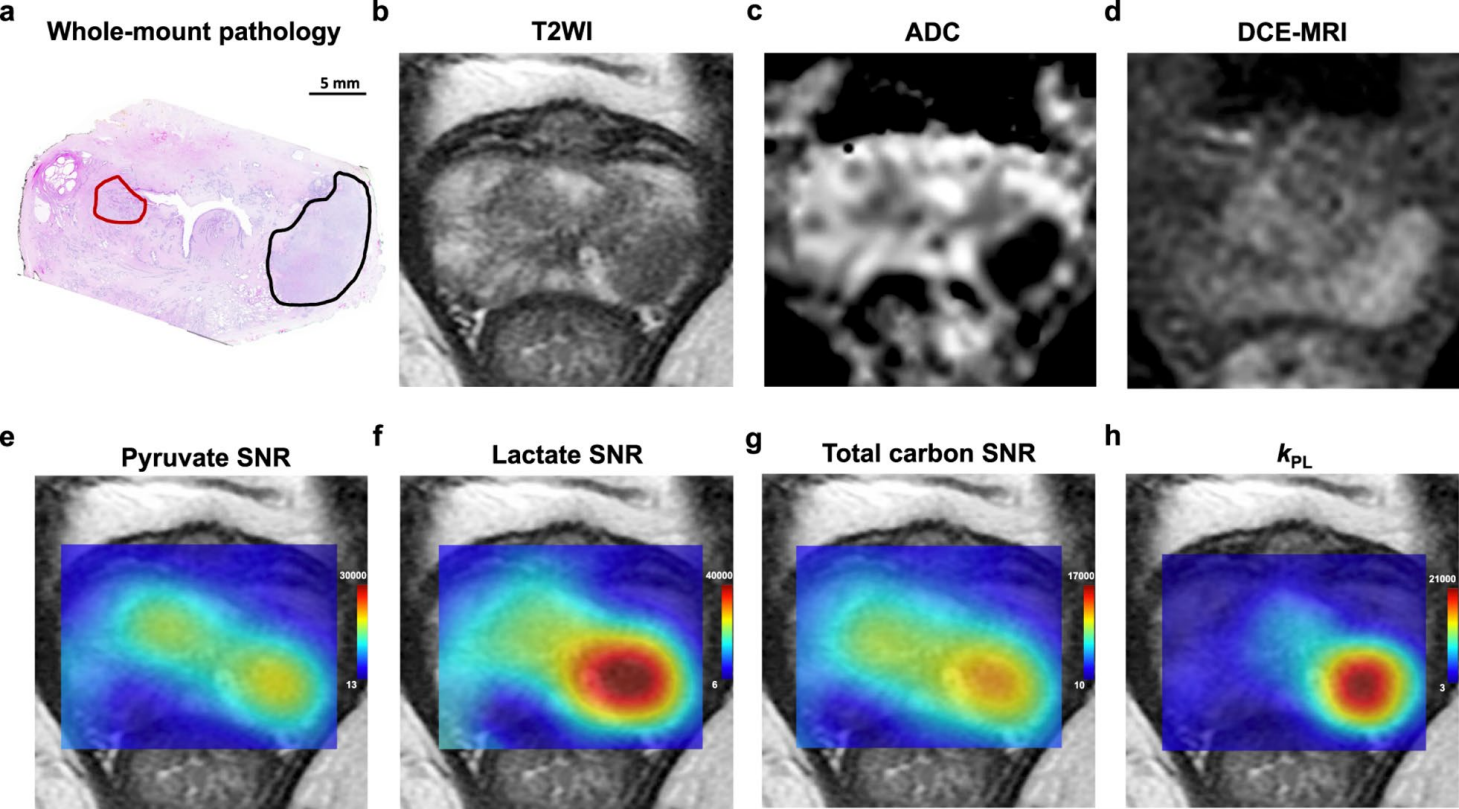
Representative example of an MR-occult transition zone tumor detected on hyperpolarized 13C-MRI. **a** Post-surgical histopathological assessment confirmed the diagnosis of multifocal adenocarcinoma of the prostate. An International Society of Urological Pathology (ISUP) grade 3 target lesion was visible on 1H-MRI in the left peripheral zone: Prostate Imaging-Reporting and Data System (PI-RADS) 5 target lesion; black region of interest (ROI). **b** Standard-of-care T2-weighted MRI demonstrating a marked area of low signal intensity corresponding to the target lesion in the left peripheral zone. **c** ADC map demonstrating a corresponding focus of markedly restricted diffusion in the left peripheral zone. **d** Dynamic contrast-enhanced (DCE) MRI demonstrating the area of early enhancement in the left peripheral zone. **e** Pyruvate signal-to-noise ratio (SNR) map with two areas of high pyruvate signal, both corresponding to histopathology-confirmed tumor foci. **f** Lactate SNR map demonstrating high [1-13C]lactate signal in the grade 3 left peripheral zone lesion. **g** Total carbon SNR map showing higher signal in the left peripheral zone tumor. **h** *K*_*pl*_ map (presented as s − 1) showing a higher rate of pyruvate-to-lactate conversion in the more aggressive left peripheral zone lesion. Nat Commun. 2022 Jan 24;13(1):466

The first clinical trials of HP-[1-^13^C]-pyruvate MRI and MRSI were successfully used in prostate cancer patients [36]. Recently, Sushentsev N et al. [37] also found that HP 13C-MRI can noninvasively differentiate indolent from aggressive prostate tumors based on their characteristic metabolic features (Fig. 5). Preclinical studies have shown that elevated [1-^13^C]-lactate is observed in animal models of prostate cancer and that the [1-^13^C]-lactate-to-[1-^13^C]-pyruvate ratio at the cancer site increased with increasing cancer stage; this ratio decreased with effective treatment [38–40]. This clinical trial study firstly demonstrated that HP-[1-^13^C]-pyruvate MRSI could be used safely and effectively in humans and secondly observed a significant [1-^13^C]-lactate peak and an increase in the [1-^13^C]-lactate-to-[1-^13^C]-pyruvate ratio in the lesion, while healthy prostate tissue and surrounding vascular tissue showed no or very low [1-^13^C]-lactate peaks [36]. This result is consistent with the results of preclinical experimental studies [38]. Albers et al. performed HP-[1-^13^C]-pyruvate MRSI in a transgenic mouse model of prostate cancer and found significant differences in the lactate content of highly differentiated prostate cancer, poorly differentiated prostate cancer, normal tissue surrounding the prostate, and metastatic lymph nodes [38]. Another noteworthy finding is that in a patient with biopsy-confirmed bilateral prostate cancer foci, conventional T2-weighted image, ADC image, and ^1^H MRSI on staging showed lesions only on the right side of the gland, while [1-^13^C]-pyruvate MRSI monitored areas of high [1-^13^C]-lactate-to-[1-^13^C]-pyruvate signal ratio on both the left and right sides of the gland, which was later confirmed by MR guided re-biopsy that confirmed a bilateral cancer focus [36]. This heralds the value of [1-^13^C]-pyruvate metabolic imaging for noninvasive tumor diagnosis, demonstrating that metabolic changes precede changes in MRI signal. In addition, the surprising finding that HP-[1-^13^C]-pyruvate MRSI can detect bilateral cancers may become a particularly important monitoring tool for patients in the slow-growing cancer category. Furthermore, for assessing tumor response to treatment, HP-[1-^13^C]-pyruvate MRSI could detect reduced pyruvate–lactate conversion early after treatment of prostate cancer [41], though low uptake and high background uptake in primary prostate cancer tissue when detected by ^18^F-FDG PET uptake imaging modalities observe the metabolic response of cancer cells more difficult [42]. Therefore, HP-[1-^13^C]-pyruvate MRSI has the potential to provide an early assessment of tumor treatment efficacy.

### Other kidney lesions

Alport syndrome (AS) is a genetic disorder characterized by impaired renal function. The development of noninvasive tools for early diagnosis and monitoring of renal function during disease progression is of clinical importance. HP-^13^C MRI is an emerging technology that allows noninvasive, real-time measurement of metabolism in vivo. A study investigated the feasibility of using this technique to assess changes in renal metabolism in a mouse model of AS. Mice with AS had significantly lower lactate levels from 4 to 7 weeks of age, whereas control mice had no change in lactate levels over time. The results of this study suggest that HP-^13^C MRI may provide a potential noninvasive tool for characterizing disease progression in AS. Furthermore, HP-^13^C MRSI was used for noninvasive assessment of oxidative stress and mitochondrial PDH activity after ischemia–reperfusion injury in the kidney. The changes in renal redox capacity and mitochondrial PDH activity on day 7 after injury in unilateral rat kidneys were observed by HP-[1-^13^C]-dehydroascorbate and^13^C-pyruvate MRSI, respectively, and it was found that on day 7 of ischemia–reperfusion injury, renal ^13^C-vitamin C-to-vitamin C plus dehydroascorbate ratio and ^13^C-bicarbonate-to-^13^C-pyruvate ratio were reduced, consistent with their low redox capacity, and PDH activity was impaired [43].

## Liver diseases

### Metabolic liver disease

A growing body of studies suggests that HP ^13^C MRI is promising for assessing liver metabolism, diagnosing liver injury by mapping the dynamic conversion of HP ^13^C substrate [44–47]. Evidences indicates that increased [1-13C]pyruvate-to-alanine-and-lactate conversion was observed in a model of chemically induced inflammatory liver injury, and the conversion of [1-13C]DHA-to-vitamin C was noted reduction in a diet-induced steatohepatitis model [2, 46, 48]. Jeong et al. found that the increased levels of alanine and lactate are useful biomarkers for fatty liver diseases. Merritt et al. studied the metabolism of HP [1-13C]pyruvate in tricarboxylic acid (TCA) cycle in rat livers and indicated that the major pathway for entry of HP [1-13C]pyruvate into the hepatic TCA cycle is via pyruvate carboxylase, and that cataplerotic flux through phosphoenolpyruvate carboxykinase (PERCK) is the primary source of [^13^C]bicarbonate [44, 49]. Another study conducted by Emine et al. [50] also showed that [^13^C]bicarbonate labeled from hyperpolarized [1- 13 C]pyruvate is an in vivo marker of hepatic gluconeogenesis in fasted state. Furthermore, the metabolic changes can be also quantified by HP ^13^C [45, 51–53]. Smith et al. [54]. measured the time to peak (TTP) of HP ^13^C-pyruvate and its metabolites in non-alcoholic fatty liver disease (NAFLD) and normal pigs, and their results revealed that the decreased liver lactate TTP in ^13^C spectra in NAFLD pigs, indicating an increased rate of lactate production and a disturbance in liver lipid synthesis. These results suggested that HP ^13^C can be used as a method for assessing in vivo hepatic metabolism and may have the potential to address a currently unmet clinical need for investigating mechanisms of fatty liver diseases (FLD), noninvasive evaluation of these pathways during the detection and treatment of FLD in general.

### Hepatocellular carcinoma

Cancer development is a multi-step process involving genetic and cellular alterations, and the multi-step hemodynamic changes for HCC during hepatocarcinogenesis have been well documented [55]. The routine used diagnostic criteria mainly refers on the vascular criteria due to the angiogenesis or neovascularization and the portal flow diminishes. However, since HCC occurs mostly within the liver parenchyma in cirrhosis, its structure is heterogeneous and manifests as multiple mass-like nodules [56]. Thus, the detection of small or very early HCC can be extremely challenging with routine imaging modalities using the vascular criteria. Using HP ^13^C MRI, Hu et al. [57] examined a model of Myc gene-induced liver cancer and found that changes in tumor metabolism precede all observable morphological and histological changes. In particularly, their results also showed that the conversion of pyruvate to alanine was significantly increased in precancerous tissue and which has become the highest alanine content region, whereas alanine was absent in healthy or established tumors. In addition to the application of early HCC detection, HP ^13^C MRI is also useful for the assessment of the biological characteristics [58, 59]. Bliemsrieder et al. [60] presented an in vivo imaging technique for metabolic tumor phenotyping in rat models of HCC, and higher pyruvate-to-lactate conversion rates and lactate signal in subcutaneous tumors were derived from high lactate-to-alanine tumor cells and which may represent a higher biological aggressiveness. Thus, metabolic alterations detected by HP ^13^C MRI provide more insights into tumor heterogeneous characteristics and therefore may act as a biological and prognostic biomarker for HCC.

### Pulmonary diseases

Currently, imaging techniques commonly used in clinical practice for the diagnosis of lung diseases include chest X-ray and computed tomography, which can obtain structural information about the lung, but they are both radioactive and cannot obtain imaging information about the gas exchange function of the lung, while usually the occurrence and development of diseases undergo a process from functional to structural lesions. Therefore, there is an urgent need to develop a non-radioactive imaging technique to detect the gas exchange function of the lung, which can be used for the early study and diagnosis of major lung diseases. Noninvasive, non-radioactive MRI has been used to obtain images of most living organs and tissues, such as the brain, heart, and abdomen, and is widely used in in vivo imaging studies and clinical disease diagnosis. However, the conventional proton (^1^H)-based MRI technique is not applicable to the lung because the lung tissue is mostly at the gas–solid interface, and thus, the magnetization rate varies greatly, resulting in a very short transverse relaxation time (T2) of protons, and even with ultrashort TE imaging sequences [61, 62], only partial information of the airways of the lung can be obtained. Moreover, the lung is composed of mostly cavernous structures, and the density of gas in the alveoli is about 1,000 times lower than that of ordinary tissues, and it is difficult to image the gas in the alveoli with conventional MRI techniques. To achieve MRI of lung gases, it is necessary to increase the polarization of the gas and thus enhance the sensitivity of the MRI signal.

**Fig. 6:**
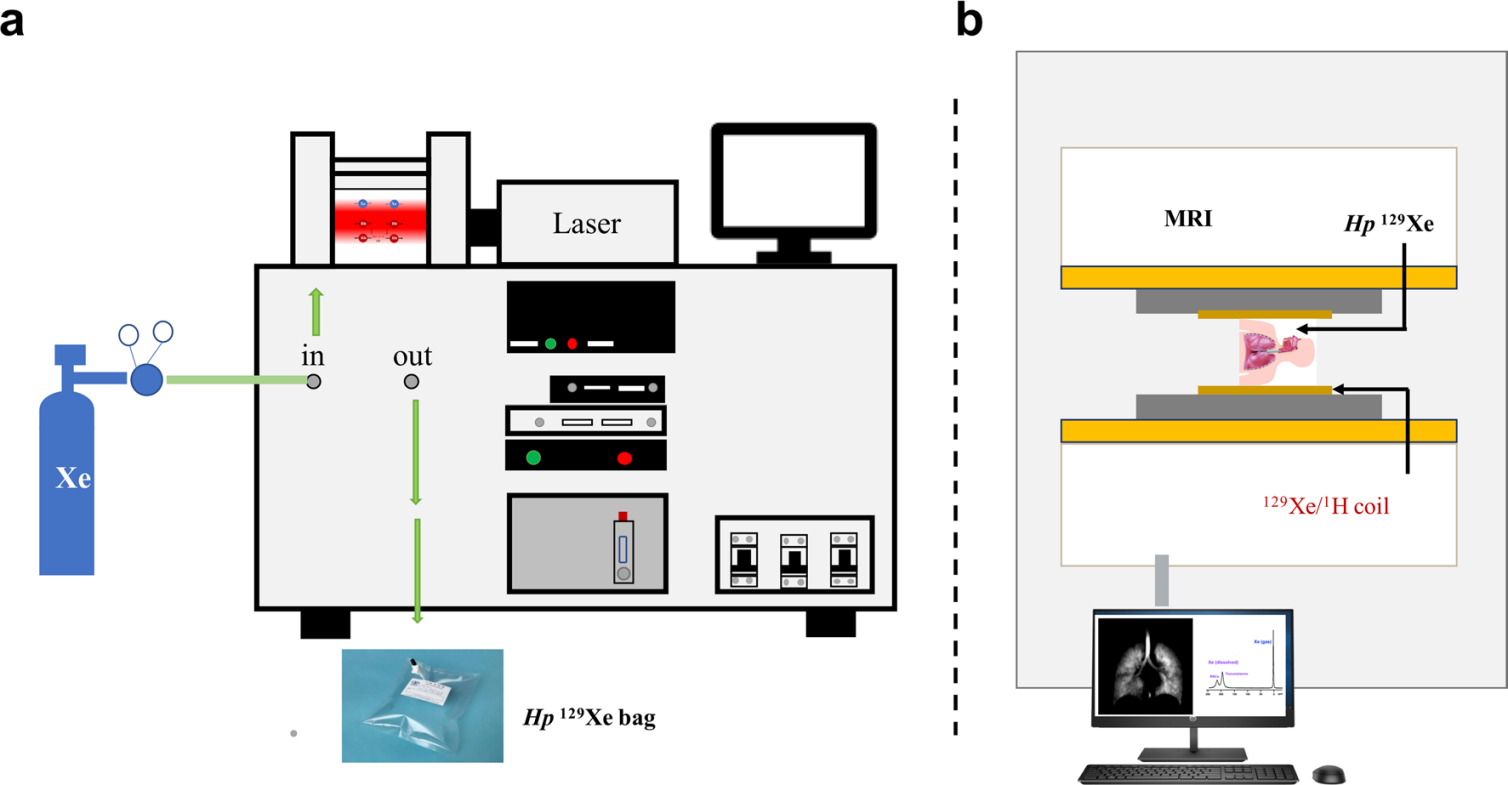
The workflow of hyperpolarized Xe 129 MRI in biomedical imaging. Preparation of the hyperpolarized Xe 129 (**a**), and the magnetic resonance imaging of the lung using Xe 129 (**b**)

**Fig. 7:**
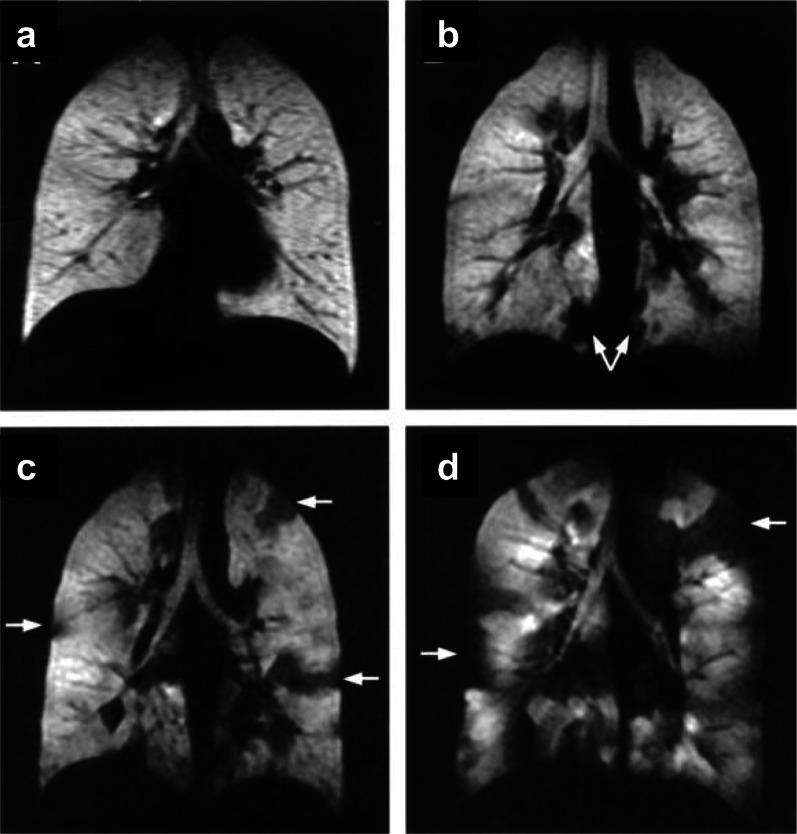
Coronal MR images obtained immediately after inhalation of hyperpolarized Helium-3 gas in a healthy normal volunteer (**a**) and in patients with mild (FEV1 of 132% of predicted value. **b** Moderate (FEV1 of 83% of predicted value. **c** Severe (FEV1 of 34% of predicted value. **d** Asthma. The distribution of the gas is homogenous in the normal volunteer, and ventilation defects are seen with increasing numbers in the asthmatic patients with increasing severity (arrows pointing at several defects). Reproduced with permission from Elsevier Ltd (License Number: 5324281420540). J Allergy Clin Immunol. 2003 Jun;111(6):1205–11

**Fig. 8:**
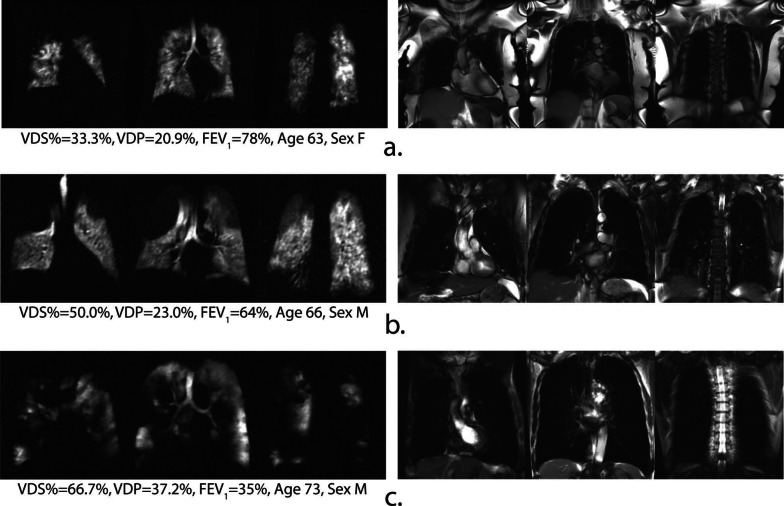
Representative 129Xe ventilation (left) and 1H SSFP anatomical image (right) slices of three COPD subjects. Note, a variety of ventilation defects score percentage (VDS%) are observed increasingly from 33.3% (**a**), 50.0% (**b**) to 66.7% (**c**) throughout the lungs. SSFP; Steady-state Free Precession. Reproduced with permission from John Wiley and Sons (License Number: 5324291003853). NMR Biomed. 2013 April; 26(4): 424–435

The noble gases (^3^He or ^129^Xe) nuclear spins are hyperpolarized, using the spin-exchange optical pumping (SEOP) technique, by several orders of magnitude compared to the Boltzmann population [63]. This leads to amplification of the MRI sensitivity by orders of magnitude compared to that of thermal equilibrium, enabling sensitive diagnosis of lung diseases by measuring associated changes in the lung's structure and function. ^3^He and ^129^Xe are presently the most used gases for HP-gas lung MRI (Fig. 6), and in recent years, HP-^83^Kr and HP-^19^F have also been reported in the literature for lung gas MRI studies [64–68]. HP-gas MRI could obtain lung images reflecting lung morphology and ventilation status, enabling visual detection of lung diseases [69–73], and HP-gas MRI could differentiate the severity of asthma [73] (Fig. 7). In combination with proton-based lung contour imaging, HP-gas MRI of lung structures can quantitatively study pulmonary ventilation disorders. Lange et al. used lung contour and ventilation information from HP-^3^He MRI to calculate ventilation deficits in healthy volunteers and patients with different degrees of asthma, and their results were not only consistent with conventional respiratory volume measurements but also allowed differentiation of asthma severity [70]. It was also possible to detect ventilation deficits in smokers with normal lung function test [71]. Since then, researchers have developed a series of semi-automated and automated algorithms and have gradually moved toward HP-^129^Xe MRI of the lungs [72] and have demonstrated that the use of HP-^129^Xe MRI and semi-automated calculations can also differentiate between healthy volunteers and patients with chronic obstructive pulmonary disease patients (Fig. 8).

**Fig. 9:**
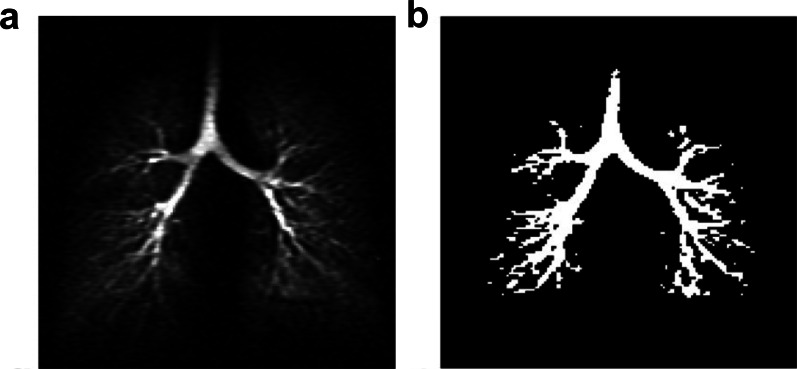
Representative dynamic coronal MR images using hyperpolarized 3He MRI. Thresholding a dynamic projection image. **a** Before thresholding; **b** After thresholding. Reproduced with permission from John Wiley and Sons (License Number: 532430065345). Reproduced with permission from John Wiley and Sons (License Number: 5324291003853). NMR Biomed. 2013 April; 26(4): 424–435

Using ultra-fast imaging sequences, HP-gas lung imaging allows not only static imaging of the lung lobes but also dynamic imaging of the trachea and bronchi of the lung [72] and obtain images of the tracheal distribution with sub-millimeter resolution [74]. Albert et al. evaluated HP-gas tracheal imaging and demonstrated that the bronchial diameters calculated using two different HP-gas imaging modalities for human class 0–5 bronchi were consistent with the classical Weibel model [75] (Fig. 9). Driehuys et al. examined healthy and partially fibrotic rats using ^129^Xe imaging and found the significant restriction of ventilation in the lesioned portion [76].

The longitudinal relaxation time T1 of HP-gas decreased sharply with increasing paramagnetic oxygen concentration in the gas [77]. After entering the lung, HP-gas mixed with oxygen remaining in the alveoli, and in areas of high oxygen concentration, the T1 of HP-gas became shorter and weaker and appeared as a low signal on ventilation imaging, so that HP-gas lung ventilation MRI can reflect the distribution of oxygen concentration. The changes in oxygen concentration in the alveoli were caused by gas exchange and ventilation in the alveoli, which can reflect lesions caused by the gas exchange in the lungs [78–81], such as chronic obstructive pulmonary disease and COVID-19 [82, 83].

The apparent diffusion coefficient (ADC) distribution of HP-gas in the lung can reflect the local structural information of the lung, which in turn can reflect the gas diffusion function in the alveoli and enable the detection of lung diseases. For example, in patients with emphysema, the alveoli became larger, and the restriction of gas movement was reduced, resulting in an increase in the ADC value of the gas in the emphysematous region [70, 84–86]. A three-channel system (^1^H, ^3^He, and ^129^Xe) could be used to simultaneously measure the diffusion coefficients of ^3^He and ^129^Xe, giving information on lung structure at different scales in a single sample [87]. In addition to the detection of lung structures, HP-gas ventilation imaging can also contribute to the diagnosis of lung cancer. Branca et al. achieved targeted detection of lung tumors by adding a superparamagnetic nanoparticle contrast agent that targets the lung tumor, affecting the homogeneity of the magnetic field in the targeted region through the superparamagnetic contrast agent, thereby altering T2* signal [88].

HP-gas MRI is non-radioactive and noninvasive and has great potential for the diagnosis and treatment of lung diseases, attracting great interest from many researchers. Although the spinning ratio of ^129^Xe is only one-third of that of ^3^He, resulting in a low MR sensitivity, the wide availability, and high natural abundance of ^129^Xe compared with the extremely scarce resources of ^3^He bring opportunities for the application of ^129^Xe MRI, especially the good lipid solubility and chemical shift sensitivity of ^129^Xe, which makes it unique for the detection of lung gas exchange function and has great potential for the early detection of lung diseases. It has great potential for the early detection of lung diseases. However, the HP-^129^Xe MRI signal sensitivity needs to be further improved to obtain higher resolution lung images and more accurate lung function information, which requires innovative methods, techniques, and instruments for generating HP-gas, optimization of HP-gas delivery systems to reduce the loss of polarization, design of new imaging coils to utilize polarization more effectively, and development of new imaging pulse sequences to obtain more functional lung information. The development of new imaging pulse sequences to obtain more functional information about the lung. Cross-innovation with clinical medicine and biochemistry is also needed for better development and application of this technology.

### Heart disease and diabetes

Phosphorus-31 is primarily used to study cell membrane phospholipid metabolism concerning ATP energy metabolism. By analyzing the concentration of phosphorus-containing metabolites in the tissue, the pH of the tissue can be obtained as well as determining the concentration of magnesium ions in the tissue [89]. ^31^P MRS can be used to measure the myocardial high-energy phosphates phosphocreatine and ATP and to determine the ratio of phosphocreatine to ATP. Calculation of the ratio has been useful in identifying ischemia in animals [90, 91] and humans with coronary stenosis [89, 92–95].

**Fig. 10:**
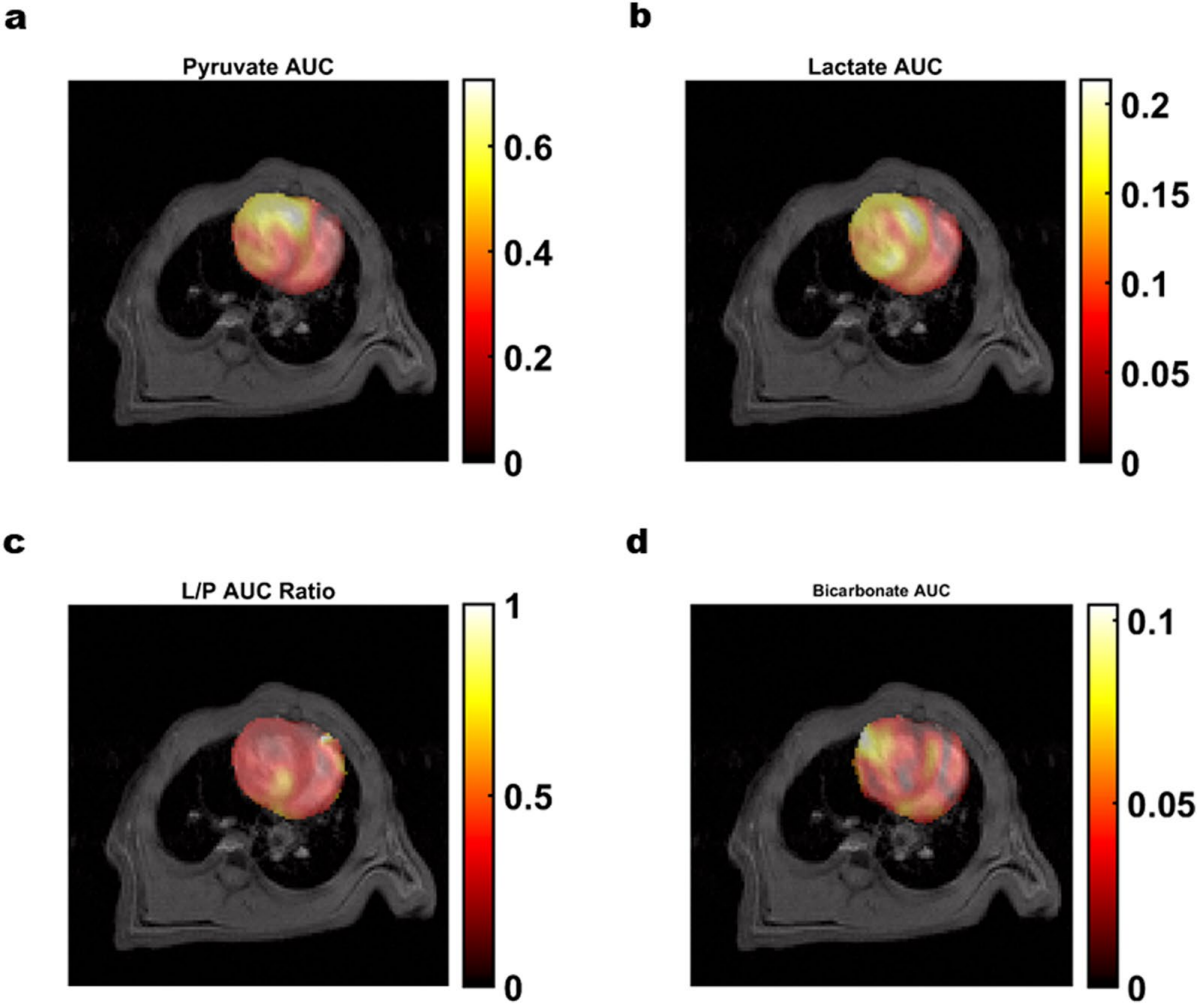
^13^C images of pyruvate (**a**), lactate (**b**), lactate/pyruvate (L/P) area under the curve (AUC) ratio (**c**), and bicarbonate (**d**) in the LV measured using dynamic HP [1-13C]pyruvate MRI. Metabolite images (color) are overlaid on T1-weighted axial images (grayscale) for anatomical reference. Reproduced with permission from Elsevier Ltd (License Number: 5324651196851). Magn Reson Imaging. 2020 May;68:9–17

Up to 40% of myocardial oxidative energy is derived from glucose and lactate in the blood, through which pyruvate is produced and enters the tricarboxylic acid cycle. HP-[1-^13^C]-pyruvate has been shown to assess cardiac PDH levels and fluxes in vitro and in vivo to provide important information on myocardial activity [96, 97]. Currently, HP MRS is mostly used to assess metabolic changes in diseases such as myocardial infarction, heart failure, cardiac hypertrophy, and cardiac tumors [98, 99] (Fig. 10).

Dodd et al. applied HP-[1-^13^C]-pyruvate and HP-[2-^13^C]-pyruvate to assess changes in mitochondrial metabolism after myocardial infarction in rats [97]. The level of acetyl coenzyme A produced via PDH at week 6 post-infarction was normal, but its oxidation was reduced (slowing of the tricarboxylic acid cycle). At week 22 post-infarction, PDH levels were found to correlate significantly with cardiac ejection fraction, and both acetyl coenzyme A production and its oxidation were reduced. This predicts that HP MRSI has important research value in the assessment of cardiac metabolism. The literature reported that HP-[1-^13^C]-pyruvate detected a significant decrease in [1-^13^C]-alanine and [1-^13^C]-lactate signals in the ischemia–reperfusion region of the left anterior descending coronary artery after 30 min of the blockade in a model of myocardial infarction, which was associated with cellular damage due to decreased cellular metabolism after severe myocardial hypoxia [100]. The HP-[1-^13^C]-pyruvate MRSI was used to determine the changes of pyruvate metabolism in the heart and liver of rats treated with metformin, and the ratio of [1-^13^C]-lactate to [1-^13^C]-pyruvate in the heart and liver increased from 0.10 to 0.27 (*p* = 0.02) and from 0.36 to 0.87 (*p* = 0.02), respectively, demonstrating that HP-[1-^13^C]-pyruvate MRSI can detect metformin-induced changes in cellular redox biology [101].

Diabetic nephropathy is one of the late complications of diabetes mellitus and one of the main causes of advanced renal failure. However, its pathogenesis remains not fully understood, and hypoxia in intrarenal tissues due to disturbances in oxygen metabolism is thought to be an important cause [102]. Laustsen C et al. used HP-[1-^13^C]-pyruvate MRI to observe alterations in renal metabolism in early-type I diabetes and found that the [1-^13^C]-lactate-to-[1-^13^C]-pyruvate ratio was significantly elevated in diabetic kidneys, whereas the [1-^13^C]-bicarbonate to [1-^13^C]-pyruvate ratio was unchanged, renal oxygen consumption was increased, and the effective oxygen consumption rate was reduced [103]. Under different oxygen concentration conditions, the metabolism of [1-^13^C]-pyruvate-to-lactate and alanine conversion in diabetic kidneys increased during acute hypoxia but did not alter bicarbonate flux, whereas lactate levels were normal at high oxygen concentrations [104]. This may explain why there is more nephropathy in diabetic patients living at high altitude [105]. Irregular insulin therapy increased the burden on the kidneys, which increased the uptake of [1-^13^C]-pyruvate and simultaneously increase the metabolic fluxes of anaerobic and aerobic metabolism (elevated lactate, alanine, and bicarbonate signals), leading to increased consumption of energy substances [106]. This suggests that strict glycemic control is important in insulin-dependent diabetes and that disturbances in renal metabolism in poor glycemic control are dependent on the consumption of energy substrates, which may herald new therapeutic targets for diabetic nephropathy.

In insulin-resistant type II diabetes, excessive hepatic gluconeogenesis and glycogenolysis can cause a rise in blood glucose, and HP-[1-^13^C]-pyruvate MRSI allowed noninvasive dynamic real-time measurement of hepatic metabolic alterations in vivo [52]. The livers of diabetic mice exhibit higher signals of ^13^C-oxaloacetate, ^13^C-aspartate, and ^13^C-malate, compared to normal mice injected with glucagon gluconeogenesis was increased similarly. Two weeks after metformin treatment, hepatic gluconeogenesis was reduced, ^13^C-aspartate and ^13^C-malate and the amount of ^13^C-labeled aspartate and malate decreased, the conversion of pyruvate to aspartate and malate also decreased, and blood glucose levels decreased, which is consistent with a downregulation of gluconeogenesis. The [1-^13^C]-pyruvate can be used as a biomarker to diagnose liver dysfunction in diabetes and to help assess treatment. The successful use of HP-^13^C metabolic imaging in diabetic disease models has a positive impact on the mechanism, treatment, and management of clinical diabetes itself and its complications.

### Challenges and future opportunities

NMR spectroscopy is the only non-radiation tool that provides the opportunity to insight the molecular metabolically changes in vivo and provides detailed structural information for molecules. NMR is thus important in real-time imaging of metabolic activities not only for biochemists but also for the clinical scientists. The exploration of the detected metabolic signals with high sensitivity, efficiency, and stability is the external pursuit for the realization of practically, clinical usefully NMR spectroscopy imaging, and this always remains the challenges. To improve the sensitivity of the NMR spectroscopy and overcome the challenges, four major directions are pushed to increase the detection sensitivity of NMR spectroscopy.

Firstly, higher magnetic fields. The sensitivity of NMR spectroscopy increases with the higher magnetic fields, and the signal of the routine used ^1^H as well as the less sensitivity nucleus such as ^2^H, ^13^C, ^15^ N, ^31^P, ^18^F, and ^23^Na will be greatly boosted with the increased magnetic fields, which provides a potential direction despite many practical difficulties. At present, many higher magnetic fields strength have been used clinically or scientifically, the cutting edge of the clinically available higher magnetic fields is 7.0 T and 9.4 T in human [107, 108], and even the much more higher 11.7 T is potentially available [109]. However, the expensive cost, massive use of liquid helium, and huge site requirements limit its transition from dedicated laboratories to widespread clinical use, and innovative design and application of newly superconducting materials may breakthrough the dilemma.

Secondly, development of high sensitivity NMR coils. Another very important strategy is to increase the signal sensitivity by developing higher sensitivity NMR coils. With the development of new materials and innovative design, more elements can be synthesized into the coil with higher density. In addition, the coil can be also designed more closely fitted with the surface of human body that means shorter coil-to-sample distance and which can also help further improve the signal sensitivity. The air coil may represent this new direction [110]. Besides, commercially available dual-tuned (^1^H and 13C) multichannel coils that allow high signal-to-noise ratio imaging for all body areas are essential [2].

Thirdly, using hyperpolarization to improve the signal sensitivity. Dynamic nuclear polarization technique has been emerged as a powerful molecular strategy which allows safe, non-radiation injury, real-time, and metabolic-specific investigation of the dynamic metabolic process. Hyperpolarized MRI of ^13^C-labeled compounds have been shown to increase the signal sensitivity more than 10,000-fold [2]; this offers great potential to trace specific metabolic process in various lesions. However, its main limitation is that the current hyperpolarization technique can be only applicable to few molecular. Generation and co-hyperpolarization of multiple molecules provide the potential to make large number of molecules to be hyperpolarized and simultaneously insight the multiple metabolic pathways. Additionally, the other limitation of the HP techniques is that the produced HP substances or HP contrast agents generally cannot be re-hyperpolarized after their administration, and the hard-won HP state will exponentially decay back to the equilibrium. This decay ostensibly poses a fundamental limit on the time scale of biochemical processes that can be probed by HP contrast agents and requires HP lifetimes that are sufficiently log (> ten of seconds) for agent manipulation after its production, administration (e.g., inhalation or injection), in vivo delivery, and observation of metabolic and functional event [111]. Thus, innovative polarization approaches should be developed to overcome the limitations of shorter hyperpolarized signal lasts time and relatively sophisticated hyperpolarization technique in the produce and probe delivery.

Last but not least, ultra-fast MR imaging sequences and innovative parsing algorithm [112]. To develop ultra-fast MR imaging sequences is another promising approach to increase the signal sensitivity. As mentioned above, the hyperpolarized signal only lasts for few minutes; thus, maximizing signal acquisition in a limited time can further increase the signal sensitivity. In addition, using innovative parsing algorithm to separate the acquired signal and to further strength the target signal may also contribute to the signal improvement.

## Conclusions

Multi-nuclear magnetic resonance spectroscopy is an emerging technique. With the development of magnetic resonance software and hardware, multi-nuclear magnetic resonance imaging has been applied to the basic and clinical transformation of various systems of human. Its unique advantages for displaying the real-time, dynamic metabolic process in different pathological processes provide the possibility for early diagnosis, evaluation of therapeutic efficacy, decision-making, drug development, and even exploration of new disease mechanisms. Despite these achievements, there is much progress yet to be made. Innovation of the polarization technique, exploration of new probes, quantification, and standardization of the results and construction of more prospective multicenter trials can further promote the clinical transformation of the advanced NMR technique.

## Data Availability

The data and material are included in this manuscript.

## Abbreviations

^18^F-FDG: ^18^F-fluorodeoxyglucose
AS: Alport syndrome
BBB: Blood–brain barrier
HP: Hyperpolarization
LDH: Lactate dehydrogenase
MCT: Monocarboxylate transporter
MP: Mononuclear phagocyte
MRI: Magnetic resonance imaging
MRSI: Magnetic resonance spectroscopic imaging
MS: Multiple sclerosis
NMR: Nuclear magnetic resonance
PDH: Pyruvate dehydrogenase
PET: Positron emission tomography
RCC: Renal cell carcinoma
TBI: Traumatic brain injury
